# Identification and expression of cuticular protein genes based on *Locusta migratoria* transcriptome

**DOI:** 10.1038/srep45462

**Published:** 2017-04-03

**Authors:** Xiaoming Zhao, Xin Gou, Zhongyu Qin, Daqi Li, Yan Wang, Enbo Ma, Sheng Li, Jianzhen Zhang

**Affiliations:** 1 Research Institute of Applied Biology, Shanxi University, Taiyuan, Shanxi 030006, China; 2 College of Life Science, Shanxi University, Taiyuan, Shanxi 030006, China; 3 Guangzhou Key Laboratory of Insect Development Regulation and Application Research, Institute of Insect Sciences and School of Life Sciences, South China Normal University, Guangzhou 510631, China

## Abstract

Many types of cuticular proteins are found in a single insect species, and their number and features are very diversified among insects. The cuticle matrix consists of many different proteins that confer the physical properties of the exoskeleton. However, the number and properties of cuticle proteins in *Locusta migratoria* remain unclear. In the present study, Illumina sequencing and de novo assembly were combined to characterize the transcriptome of *L. migratoria*. Eighty-one cuticular protein genes were identified and divided into five groups: the CPR family (51), Tweedle (2), CPF/CPFLs (9), CPAP family (9), and other genes (10). Based on the expression patterns in different tissues and stages, most of the genes as a test were distributed in the integument, pronotum and wings, and expressed in selected stages with different patterns. The results showed no obvious correlation between the expression patterns and the conservative motifs. Additionally, each cluster displayed a different expression pattern that may possess a different function in the cuticle. Furthermore, the complexity of the large variety of genes displayed differential expression during the molting cycle may be associated with cuticle formation and may provide insights into the gene networks related to cuticle formation.

The main function of arthropod cuticle is composed exoskeleton, which plays an important role in keeping the body structure, inhibiting the evaporation of water and serving as a barrier to the environment. According to several morphologically of the exoskeleton, the insect molt cycle is composed of four distinct stages: pre-molt, ecdysis, post-molt, and inter-molt. In the period of pre-molt (apolysis), the old cuticle is separated from the underlying epidermis, and partially digested and reabsorbed. During the course of ecdysis, new epicuticle and exocuticle are secreted and formed, and then the old exoskeleton is shed. In the period of post-molt stage, the partially formed new exoskeleton expanded, the pre-ecdysial layers take place tanning and sclerotization, meanwhile the endocuticle is deposited and become hardened. And a mature exoskeleton is formed at the inter-molt stage^1^.

Insect cuticle layers are composed of many types of cuticular proteins interact with chitin^2^. The sequences of more than seven hundred cuticular proteins are available from the cuticleDB website^3^ (http://bioinformatics2.biol.uoa.gr/cuticleDB/index.jsp), because they have been identified from numerous insect species and several other arthropods. Most of the cuticular proteins have the Rebers and Riddiford Consensus (R&R Consensus), which contains the chitin binding domain (ChtBD) and binds chitin^4^,^5^,^6^. It has been classified as belonging to the CPR protein family for this proteins that contain the R&R Consensus. The CPR protein family was further divided three groups, RR-1, RR-2, and RR-3, which are related to the type or region of the cuticle. There is a tentative classification, CPRs with the RR-1 type domain have been considered as contributing to soft (flexible) cuticles, whereas RR-2 proteins have been associated with rigid (hard) cuticles^6^,^7^. Besides, RR-3 protein was also proposed^7^, but a precise definition has not been established^8^. As reported by Jasrapuria *et al*.^9^, 39 genes were predicted from *Tribolium castaneum*, which encode two different families of proteins with ChtBD2 motifs, called “Cuticular Protein Analogous to Peritrophins” (CPAPs). In *T. castaneum*, the expression of CPAP family genes was detected exclusively in epidermal tissues, and not in midgut^9^. Based on whether these proteins contain either one or three ChtBD2 domains, they have been classified into two families, CPAP1 and CPAP3 family, respectively. The genes encoding the CPAP3 family of proteins are the ortholog of the “gasp” or “obstructor” genes previously reported in *Drosophila melanogaster*^10^,^11^. Many of these proteins play a fundamental and indispensable role in maintaining the structural integrity of the cuticle in different parts of the insect anatomy^9^.

Recently, researchers have reported many cuticular proteins with different motifs, such as Tweedle, CPF (cuticular protein with a 44 amino acid motif) and CPF-like proteins (CPFL). The Tweedle motif was identified from a body shape mutant in *D. melanogaster*^12^. Secondary structure of Tweedle proteins with the consensus motif were previously predicted and showed a preponderance of β-pleated sheet with distinct strands in these proteins from cuticle, and in this case, aromatic residues (tyrosine and phenylalanine) were found on one face within a sheet, which provide an optimal location for interaction with chitin^13^,^14^. In *D. melanogaster*, studies demonstrated that a mutation of a Tweedle protein alters the body shape, and noted that several of highly conserved residues located within the β-strands, which might be hypothesized the Tweedle family proteins could interact directly with chitin^12^. For CPF proteins, a 51-residue conserved region was first identified from six cuticular proteins of *Tenebrio molitor* and *Locusta migratoria*^15^. However, more recently, Togawa *et al*. held the view that the conserved region is only 44 amino-acids long when more species were examined^16^. Different from CPR family proteins, two CPF recombinant proteins did not bind chitin *in vitro*^16^. CPFL proteins were also identified, which have similar C-terminal regions with that of CPF proteins, but lack the conserved 44 amino-acids residues. By now, more and more cuticular proteins have been isolated and sequenced directly from cuticles using proteomics analysis, and different family cuticular proteins have different conserved motifs^2^,^6^,^17^.

After the first insect genome, *D. melanogaster*, was sequenced^18^, more than one hundred insect genome sequences are now available at NCBI. Based on these genome sequences, cuticular proteins with an R&R motif have been exhaustively identified in *D. melanogaster*^8^ and *Apis mellifera*^19^. From these, 101 and 28 cuticular proteins containing the R&R motif were identified, respectively. In *Bombyx mori*, 220 putative cuticular protein genes were found by a genome-wide screen, including RR-1 (56), RR-2 (89), RR-3 (3), Tweedle (4), CPF (1), CPFL (4), glycine-rich (29), and other genes (34)^20^. Phylogenetic analysis using RR-1 and RR-2 proteins from *B. mori, D. melanogaster*, and *A. mellifera* showed that duplicate cuticular protein clusters have evolved independently among insect taxa^20^, in other words, the composition of cuticular protein genes may be unique among insect taxa.

The migratory locust, *L. migratoria*, is the most destructive agricultural pests which has long served as a model organism for many aspects research, such as insect morphology, behavior and physiological^21^,^22^,^23^. However, little is known regarding the cuticular protein genes of *L. migratoria*. In the present study, Illumina sequencing and de novo assembly were combined to obtain and characterize the transcriptome of the different developmental stages of *L. migratoria*. In total, 4.82 Gb nucleotides were generated, and 84,641 Unigenes were assembled from the *L. migratoria* whole-body library. To identify cuticular protein genes in *L. migratoria* exhaustively, we searched the whole-body library sequences and found 262 Unigenes annotating cuticular protein genes. We then annotated again and deleted the repeat genes. Finally, 81 cuticular protein genes were identified, including RR-1 (25), RR-2 (18), RR-3 (8), Tweedle (2), CPF/CPFLs (9), CPAPs (9), and other genes (10). Additionally, we performed reverse-transcription PCR (RT-PCR) and reverse-transcription quantitative PCR (RT-qPCR) analysis to determine the expression profiles of several key cuticular protein genes from different families in different tissues and different stages of *L. migratoria*. All of these results provide valuable information to analyze the role of cuticular protein genes involved in insect development and cuticle formation during ecdysis.

## Results

### Illumina sequencing and data assembly of *L. migratoria* transcriptome

To obtain more detailed information regarding the cuticle protein genes of *L. migratoria*, cDNA library from the whole body at different stages was sequenced using the Illumina HiSeq2000 sequencing platform. After the cleaning of dirty reads and quality checks, 53,559,770 high-quality clean reads with a cumulative length of 4,820,379,300 nucleotides (4.82 Gb) were generated from the whole-body library (Fig. S1A). The GC percentage of the reads was 46.44% (Fig. S1A), which is comparable to the genome sequence of other insects. These reads were assembled into 188,554 contigs with an average length of 260 nt (contig N50 was 352 nt) (Fig. S1B). These contigs were further assembled into 84,641 Unigenes longer than 200 nt (average size 491 nt and N50 was 692 nt) using paired end-joining and gap-filling (Fig. S1B). The size distribution indicated that 89.61% of Unigenes was in 200–1000 nt, and that the lengths of the 8794 (10.39%) Unigenes were above 1000 nt (Fig. S1C), which was a significantly greater percentage than that found previously in insect transcriptome projects^24^,^25^. This Transcriptome Shotgun Assembly project has been deposited at DDBJ/EMBL/GenBank under the accession GEZB00000000. The version described in this paper is the first version, GEZB01000000.

### Functional annotation and classification of predicted proteins

To uncover the molecular events underlying the transcriptomic profile, all Unigene sequences were aligned to the protein databases, including nr, Swiss-Prot, KEGG, GO and COG (E-value < 0.00001) using BLASTX, and nucleotide database nt (E-value < 0.00001) using BLASTN. The Unigenes along with their functional annotations were retrieved with the highest sequence similarity to proteins. Of the 84,641 Unigenes, we found that 26,696 (31.54%), 12,130 (14.33%), 21,327 (25.2%), 18,674 (22.06%), 9,160 (10.82%), and 13,304 (15.72%) were annotated in nr, nt, Swiss-Prot, KEGG, COG, and GO, respectively (Table S3). Overall, 29,289 Unigenes (34.6%) were annotated to at least one database. There were still 55,352 Unigenes (65.4%) that not matched to any databases mentioned above. As shown in the results, the annotation percentage is low. One possibility is that the transcripts derived from the untranslated regions, misassembled contigs or non-conserved domains can’t be annotated^25^. Additionally, it could be speculated that a large part of the genes in *L. migratoria* transcriptome database are with unknown functions or the potential novel genes.

**Figure 1:**
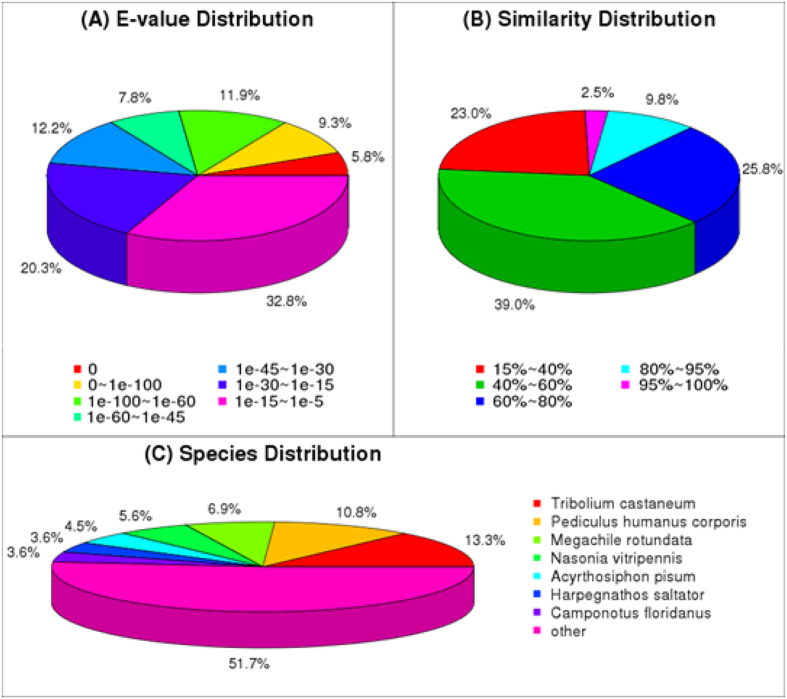
Homology analysis of *L. migratoria* Unigenes. (**A**) E-value distribution. (**B**) Similarity distribution. (**C**) Species distribution. All Unigenes that had BLASTX annotations within the NCBI nr database with a cut-off E-value of 10^−5^ were analyzed. The first hit of each sequence was used for analysis.

As shown in Fig. 1, among the annotated Unigenes, 7,208 Unigenes (approximately 27%) showed a high homology (E-value < 1e-60) which specifically matched this database (Fig. 1A). The identity comparison showed 10,171 (38.1%) Unigenes have more than 60% identity with other insects (Fig. 1B). The top 7 species distributions are shown in Fig.1C. Approximately 12,894 Unigenes (48.3%) were annotated to 7 top-hit insect species. *T. castaneum* and *Pediculus humanus corporis* were the 2^nd^ top-hit species, with 3,551 (13.3%) and 2,883 (10.8%) annotated genes, respectively. The other top-hit species were *Megachile rotundata* (6.9%), *Nasonia vitripennis* (5.6%), *Acyrthosiphon pisum* (4.5%), *Harpegnathos saltator* (3.6%), *Camponotus floridanus* (3.6%) (Fig. 1C). The functional classification of all Unigenes was predicted by performing GO analyses. A total of 84686 Unigenes were allocated to three specific GO categories: cellular component, biological process and molecular function. Among them, approximately 40,947 Unigenes (50.89%) at the biological process level, 26,253 Unigenes (27.42%) at the cellular component level and 17,486 Unigenes (21.69%) at the molecular function level. In total, 51 categories were subdivided from the subcategories: 25 subcategories for biological process, 15 subcategories for cellular component and 11 subcategories for molecular function. Among these subcategories, cellular processes (18.98%) and metabolic processes (15.52%), cell (22.28%) and cell part (22.27%), and binding (40.88%) and catalytic activities (39.64%) were the most abundant under the biological process, cellular component and molecular function, respectively (Fig. S2). The subcategories which were the two largest proportion in each category were similar to that of other species studied previously^26^,^27^. For COG functional classification of the Unigenes, approximately 9,160 Unigenes were involved in 25 COG categories (Fig. S3). Among them, the largest group was the “General function prediction only” (3,607 Unigenes, 39.4%), followed by the large groups (i.e., > 1500 Unigenes) “Function unknown” (1,688 Unigenes, 18.4%), “Replication, recombination and repair” (1,687 Unigenes, 18.4%), “Translation, ribosomal structure and biogenesis” (1,645 Unigenes, 18%), “Transcription” (1,623 Unigenes, 17.7%) (Fig. S3).

### Identification and comparison of cuticle protein genes in *L. migratoria*

**Figure 2:**
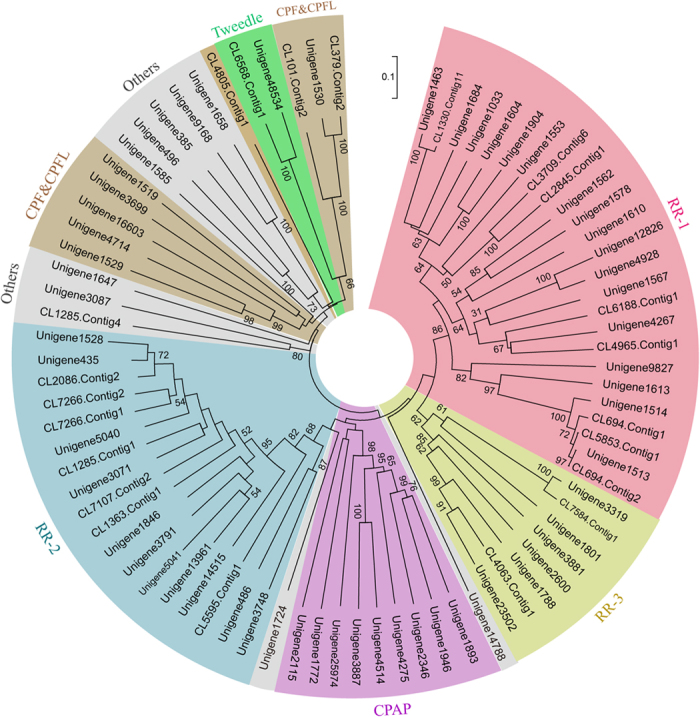
The neighbor-joining tree of all cuticle proteins based on *L. migratoria* transcriptome. Phylogenetic tree was constructed with the neighbour joining method of MEGA 5 using the pairwise deletion of indels. Bootstrap support is based on 1000 resembled data sets. The different color indicate RR-1, RR-2, RR3, CPAPs, CPF/CPFLs, Tweedle and other genes, respectively. The classification of cuticle proteins was listed in Table S4.

To identify cuticular protein genes in *L. migratoria* exhaustively, we screened the whole body library sequence with known motifs such as R&R Consensus for CPR family, Tweedle motif for Tweedle genes, CPAP1 and CPAP3 sequences for CPAPs family, and 44 amino-acids residues or AAP(A/V) for CPF/CPFLs. We found 262 Unigenes that annotated cuticular protein genes from *L. migratoria* transcriptome. Next, we annotated again and deleted the repeat genes, finally identifying 81 cuticular protein genes. The classification was confirmed by an HMM tool in the cuticleDB website^8^. Of these, 25 and 18 were grouped as RR-1 and RR-2 proteins, respectively (Table 1). We identified two *Tweedle* genes with a Tweedle motif and nine CPF/CPFL genes with the 44 amino-acids consensus or AAP(A/V) motif. According to the CPAP family of *T. castaneum*, we have identified two CPAP1 family and seven CPAP3 family cuticle proteins that contain one or three ChtBD2 domains, respectively. However, the remaining ten genes annotating cuticle proteins do not belong to any family because they do not have the motifs described above (Table 1). Using phylogenetic analysis with the neighbor-joining method, five main groups of cuticular protein were identified as follows: CPR family containing RR-1 (30.9%), RR-2 (22.2%), RR-3 (9.9%), CPAPs (11.1%), CPF/CPFLs (9.9%), Tweedle (2.5%) and other proteins (13.5%) (Fig. 2). The Unigene ID, and Family and Gene descriptions of these genes are denoted in Table S4.

**Figure 3:**
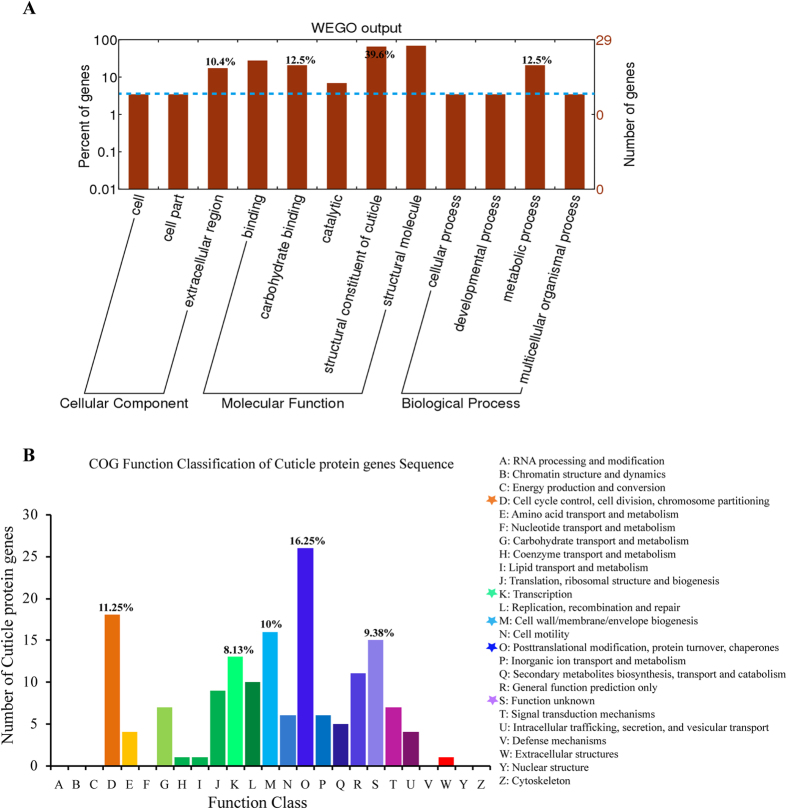
Gene ontology (GO) assignment and Clusters of orthologous groups (COG) classification of *L.* *migratoria* cuticle protein genes. (**A**) The GO classification map was done by uploading the GO ID numbers of genes for their involvement in biological processes, cellular components, and molecular functions. The number of Unigenes assigned to each GO term is shown in the right column. (**B**) A total of 81 produced functional annotations were among the 25 categories. The Y-axis shows the number of cuticle protein genes in each COG term.

Next, all of the cuticle protein genes as described above were further assigned to GO classification for the potential functions. As shown in Fig. 3, extracellular region (10.4%) was the largest sub-category of cellular component. In the molecular function category, structural constituent of cuticle (39.6%) was the most abundant subcategory, followed by carbohydrate binding (12.5%). The biological process assignments were mostly dominated by the group of metabolic processes (12.5%) (Fig. 3A). Moreover, to further predict the putative cuticular protein functions, a COG analysis was performed. The results showed the cluster for “Posttranslational modification, protein turnover, chaperones” (16.25%) constituted the largest group, followed by “Cell cycle control, cell division, chromosome partitioning” (11.25%), “Cell wall/membrane/envelope biogenesis” (10%), “Function unknown” (9.38%) and “Transcription Translation” (8.13%) (Fig. 3B).

### Identification and expression analysis of the CPR family genes in *L. migratoria*

**Figure 4:**
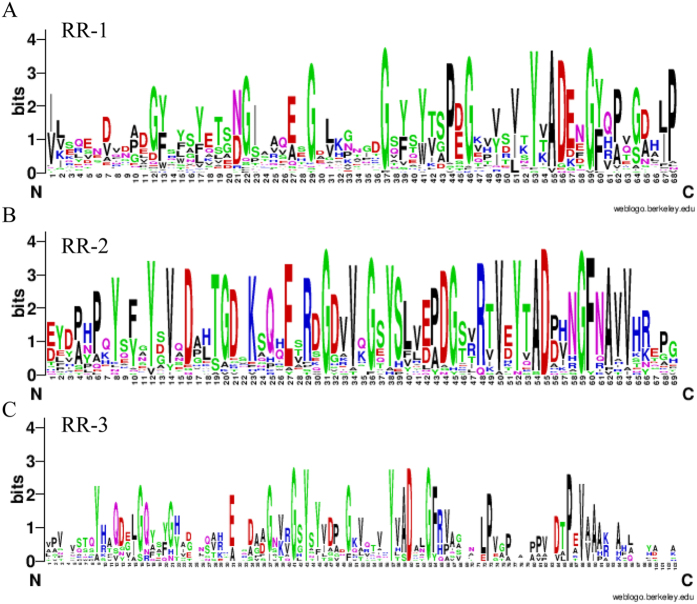
Motif identification of CPR genes. RR motifs were shared by 42, 29 and 13 genes from Unigenes and other species that annotated CPR genes, respectively. The Weblogo online sever was used to identify the common elements in CPR genes (http://weblogo.berkeley.edu/logo.cgi). (**A**–**C**) RR-1, RR-2 and RR-3, respectively.

In the present study, we found 51 CPR cuticle proteins from the *L. migratoria* transcriptome with R&R motifs, including RR-1 (25), RR-2 (18) and RR-3 (8). The R&R Consensus in the key site of amino acids is conserved, consistent with previous research (Fig. 4A–C), and the same as that of the other insects. However, the amino acid sites vary considerably besides the RR motif.

**Figure 5:**
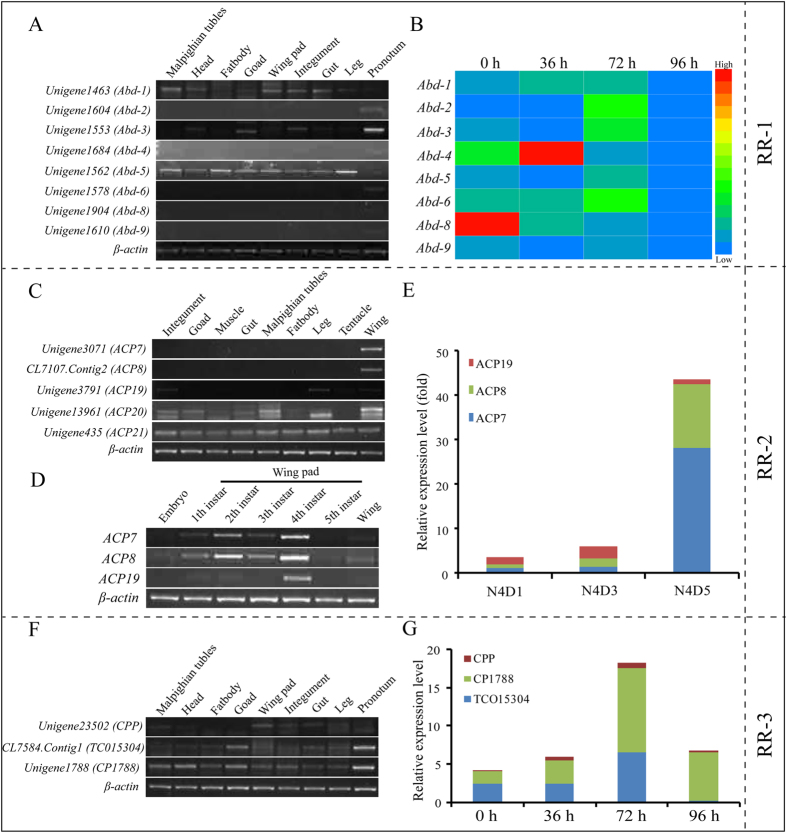
Expression profiles of the members of the CPR gene families (RR1, RR2 and RR3) as determined by RT-PCR and RT-qPCR. (**A**) Expression of RR-1 gene-annotated endocuticle protein genes in different tissues on day 6 of 5^th^ instar nymphs, as detected by RT-PCR (28 cycles). Different tissues: Integument, head, leg, pronotum, goad, gut, Malpighian tubules, fat body, wing pad; (**B**) Expression of RR-1 genes in the pronotum after molting from 4^th^ instar nymphs to 5^th^ instar nymphs at 0 h, 36 h, 72 h and 96 h, as detected by RT-qPCR. Heat map showing the relative expression level during different stages of RR-1 genes. The colors in the map display the relative values of all tiles within the 4 given developmental stages. Blue indicates the lowest expression, green indicates intermediate expression, and red indicates the highest expression. The color scale bar is shown on the right of the figure. (**C**) Expression of RR-2 gene-annotated adult cuticle protein genes in different tissues on day 2 of adults as detected by RT-PCR (28 cycles). Different tissues: Integument, leg, muscle, tentacle, goad, gut, Malpighian tubules, fat body, wing. (**D**) Expression of RR-2 genes in whole body, wing pad or wing from embryo, 1, 2^nd^, 3^rd^, 4^th^, 5^th^ instar nymphs to the adults as detected by RT-qPCR. (**E**) Expression of RR-2 genes in the wing of 5^th^ instar nymphs at different days as detected by RT-qPCR. N4D1, N4D3, N4D5: Day 1, Day 3, Day 5 of 5^th^ instar nymphs. (**F**) Expression of RR-3 genes in different tissues on day 6 of 5^th^ instar nymphs as detected by RT-PCR (28 cycles). Different tissues: Integument, head, leg, goad, gut, Malpighian tubules, fat body, wing pad, pronotum; (**G**) Expression of RR-3 genes in the pronotum after molting from 4^th^ instar nymphs to 5^th^ instar nymphs at 0 h, 36 h, 72 h and 96 h, as detected by RT-qPCR. *β-actin* was used as the reference control for RT-PCR (24 cycles) and RT-qPCR, respectively. All data are reported as means ± SE of three independent biological replications. The electrophoresis image were obtained by using the gel imaging analysis system (Bio-Rad, USA). The full-length gels are presented in Supplementary Figs 1–4, Figs 2–4 and Figs 3–4, respectively.

Among them, we found a cluster of Unigenes with an RR-1 motif that annotated the endocuticle structural glycoprotein found in the abdomen (*Abd*) of *Schistocerca gregaria*^28^,^29^. Almost all these proteins were forecasted to have a cleavable signal peptide, and all of them contained a chitin binding domain, which suggested that they were secreted and able to interact with extracellular chitin potentially. The results of tissue expression by RT-PCR showed that Unigene1463 (*Abd-1*) and Unigene1562 (*Abd-5*) were expressed in all of the tested tissues; additionally, with a high expression level in pronotum, Unigene1553 (*Abd-3*) was also expressed in the head, integument, goad and gut. However, Unigene1604 (*Abd-2*), Unigene1684 (*Abd-4*), Unigene1578 (*Abd-6*), Unigene1904 (*Abd-8*) and Unigene1610 (*Abd-9*) were only highly expressed in the pronotum, not in other tissues (Fig. 5A). In order to further validate the results of these genes, we again detected the expression of these genes by adjusting the cycle number (32 cycle). As shown in Fig. S4–1, the genes of Unigene1684 (*Abd-4*), Unigene1904 (*Abd-8*), and Unigene1610 (*Abd-9*) similarly have a higher expression in pronotum, but also a lower expression in leg or integument. In addition, we also detected the expression of Unigene1604 (*Abd-2*) and Unigene1578 (*Abd-6*), and the results showed they mainly expressed in pronotum and integument, little in head, goad, wing pad and leg under these conditions.

According to the developmental expression patterns in the 5^th^ instar nymphs of *L. migratoria*, the expression of Unigene1463 (*Abd-1*), Unigene1578 (*Abd-6*) and Unigene1684 (*Abd-4*) was gradually increased after molting, was up to the highest level at 36 h and 72 h, respectively, and then decreased gradually (Fig. 5B). This expression pattern was consistent with the formation of endocuticle, which suggest they may be involved in the synthesis of endocuticle. We also found Unigene1604 (*Abd-2*), Unigene1553 (*Abd-3*), Unigene1562 (*Abd-5*) and Unigene1610 (*Abd-9*) had a high expression level at 0 h and 72 h after the molting stage of the 5^th^ instar nymphs, but Unigene1904 (*Abd-8*) had the highest expression level at 0 h after the molting stage and then gradually decreased from 36 h to 96 h (Fig. 5B). It suggested that Unigene1904 (*Abd-8*) was consistent with the formation of exocuticle and might be involved in the synthesis of exocuticle in *L. migratoria.*

For the expression profiles of RR-2 motif cuticle protein genes, we selected five Unigenes that annotated the adult cuticle protein gene (*ACP*) from cuticleDB, as detected by RT-PCR and RT-qPCR. The results showed that Unigene3071 (*ACP7*) and CL7107. Contig2 (*ACP8*) were specifically expressed in the wings at the transcript level, Unigene3791 (*ACP19*) was highly expressed in the wings, integument and leg, while Unigene13961 (*ACP20*) and Unigene435 (*ACP21*) were expressed in almost all of the tested adult tissues (Fig. 5C). To explore their expression pattern at different stages, the mRNA levels from the whole body of embryos, 1 instar nymphs, 2^nd^ instar nymphs, the wing pads of 3^rd^–5^th^ instar nymphs and the wings of adults were analyzed using RT-PCR. The results showed that Unigene3071 (*ACP7*), CL7107.Contig2 (*ACP8*) and Unigene3791 (*ACP19*) were highly expressed in 4^th^ instar nymphs (Fig. 5D). Unigene3071 (*ACP7*) and CL7107.Contig2 (*ACP8*) had the highest expression level at day 5 of 4^th^ instar nymphs as examined by RT-qPCR, whereas Unigene3791 (*ACP19*) was highly expressed at day 3 of 4^th^ instar nymphs (Fig. 5E). Thus, Unigene3071 (*ACP7*) and CL7107.Contig2 (*ACP8*) might have different functions from those of Unigene3791 (*ACP19*).

The cuticle proteins with RR-3, which is a small family, were annotated as hypothetical proteins. To verify the tissue and stage specificity of the gene expression, gene expression was examined in different tissues at day 6 of the 5^th^ instar using RT-PCR. The results revealed higher expression in the pronotum, lower expression in other tissues and no display of tissue specificity (Fig. 5F). The expression levels of all of the genes were gradually increased after the molting, reached the highest at 72 h, and then decreased gradually as examined by RT-qPCR (Fig. 5H). Their expression pattern was also consistent with the formation of endocuticle, indicating they probably participated in the synthesis of endocuticle.

### Identification and expression analysis of CPAP family genes in *L. migratoria*

**Figure 6:**
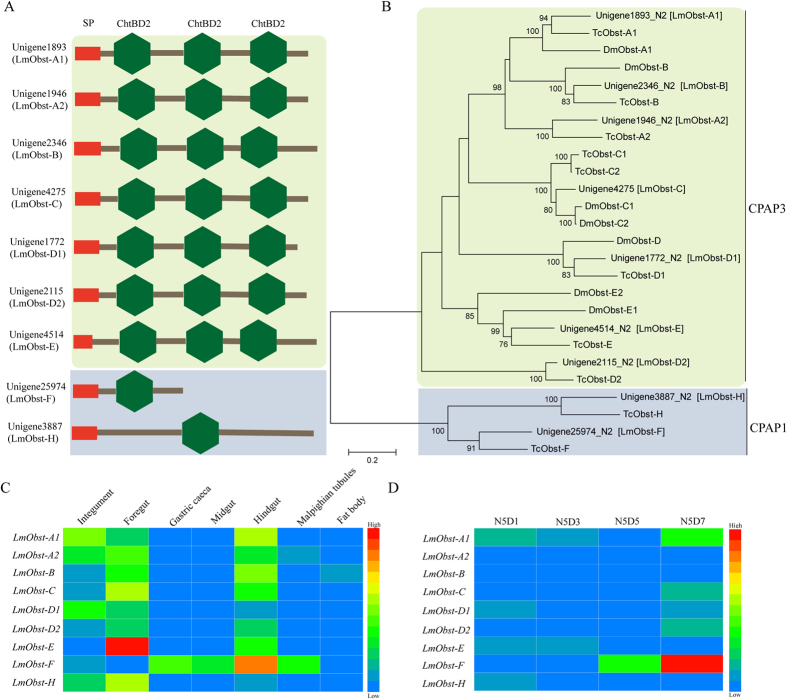
Structural, Neighbor-joining tree and Expression profiles of members of the CPAP gene families (CPAP1 and CPAP3). (**A**) The structure of CPAP cuticular proteins. Red indicates the predicted signal peptide, green indicates the chitin-binding peritrophin-A domain (ChBD2). (**B**) A phylogenetic tree was constructed with the neighbor-joining method of MEGA 5 using the pairwise deletion of indels. Bootstrap support was based on 1,000 resampled data sets. The GenBank accession numbers are listed in Table S1. (**C**) Expression of CPAP1 and CPAP3 in different tissues on day 6 of 5^th^ instar nymphs as detected by RT-qPCR. Different tissues are listed: Integument, foregut, midgut, gastric caeca, hindgut, Malpighian tubules, fat body; (**D**) Expression of CPAP1 and CPAP3 in the integuments of 5^th^ instar nymphs at different days as detected by RT-qPCR. N5D1, N5D3, N5D5, N5D7: Day 1, Day 3, Day 5, day 7 of 5^th^ instar nymphs. *β-actin* was used as the reference control. All data are reported as means ± SE of three independent biological replications. Heat map showing relative expression level during different tissues and stages of nine CPAP cuticular protein genes. The colors in map display the relative values of all tiles within the given 7 different tissues or 4 developmental stages. Blue indicates the lowest expression, green indicates the intermediate expression, and red indicates the highest expression. The color scale bar is shown on the right of the figure.

According to the sequences of CPAPs in *T. castaneum* and Obstruct in *D. melanogaster*, we obtained nine CPAP genes, including two CPAP1 and seven CPAP3 genes, from the transcriptome database of *L. migratoria* and obtained their full-length sequences based on the genomic of database *L. migratoria*^30^. The sequences were submitted to NCBI, and the accession numbers are listed in Table S1. They all have a signal peptide, three ChtBD2 for CPAP3, and one ChtBD2 for CPAP1 (Fig. 6A). Phylogenetic tree analysis showed that they were clustered into the subclass of *D. melanogaster* or *T. castaneum*, respectively (Fig. 6B). Different tissue expression showed that they were highly expressed in the tissues derived from the ectoderm such as the foregut, hindgut or integument at day 6 of 5^th^ instar nymphs by RT-qPCR, except for Unigene3887 (*LmObst-F*), which was highly expressed in the gastric caeca, midgut, hindgut, and Malpighian tubule but had a low expression level in other tissues (Fig. 6C). Based on the results of developmental stage expression, Unigenes *LmObst-D1, LmObst-E* and *LmObst-H* were highly expressed at day 1 of 5^th^ instar nymphs (after molt from 4^th^ instar nymphs), whereas other Unigenes (*LmObst-A1, LmObst-A2, LmObst-B, LmObst-C, LmObst-D2* and *LmObst-F*) were highly expressed at day 7 of 5^th^ instar nymphs (before molting) (Fig. 6D). The results suggested that CPAP genes have a different expression pattern, although they are clustered in the same family, implying that they probably play different functions during the formation of cuticle in *L. migratoria*.

### Identification and expression analysis of non-chitin-binding domain (CPF/CPFL, Tweedle family and others) genes in *L. migratoria*

**Figure 7:**
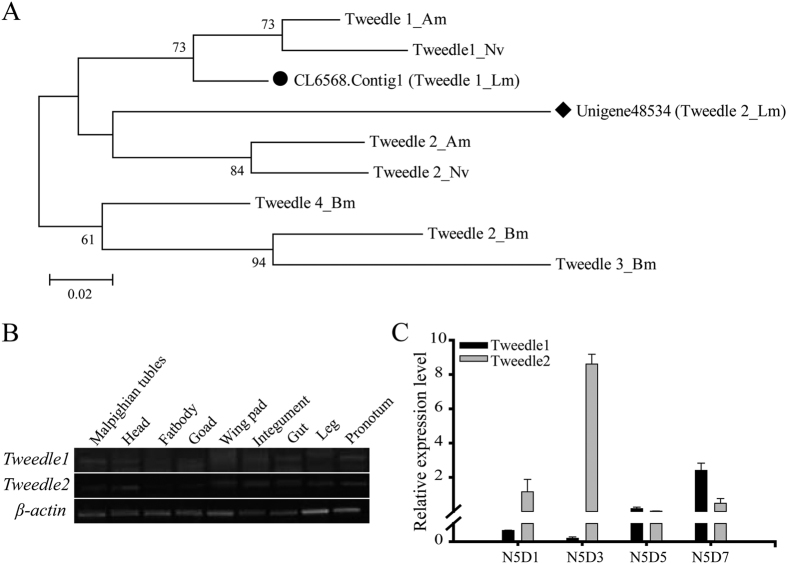
Neighbor-joining tree and Expression profiles of members of the Tweedle gene families. (**A**) A phylogenetic tree was constructed with the neighbor-joining method of MEGA 5 using the pairwise deletion of indels. Bootstrap support is based on 1000 resembled data sets. GenBank accession numbers are listed in Table S1. (**B**) Expression of Tweedle genes in different tissues on day 6 of 5^th^ instar nymphs as detected by RT-PCR (28 cycles). *β-actin* was used as the reference control (24 cycles). Different tissues are listed: Integument, head, leg, goad, gut, Malpighian tubules, fat body, wing pad, pronotum; The image were obtained by using the gel imaging analysis system (Bio-Rad, USA). The full-length gels are presented in Supplementary Fig. 5. (**C**) Expression of Tweedle genes in the pronotum of 5^th^ instar nymphs at different days as detected by RT-qPCR. N5D1, N5D3, N5D5, N5D7: Day 1, Day 3, Day 5, day 7 of 5^th^ instar nymphs. *β-actin* was used as the reference control. Data are reported as means ± SE of three independent biological replications.

In the transcriptome database of *L. migratoria*, we searched two Tweedle protein genes (CL6568.Contig1, *Tweedle1* and Unigene48534, *Tweedle2*) with the Tweedle motif. Using phylogenetic tree analysis, they form a cluster with the Tweedle1 and Tweedle2 protein of *Apis mellifera* and *Nasonia vitripennis*, respectively (Fig. 7A). Tissue expression analysis by RT-PCR showed that the two Tweedle protein genes have a higher expression level in pronotum, but no tissue specificity (Fig. 7B). Different developmental stage expression showed that *Tweedle1* had a higher expression level at day 7 of the 5^th^ instar nymphs, while *Tweedle2* was at day 3 (Fig. 7C). The two genes have different expression patterns, implying that their function may be distinct.

**Figure 8:**
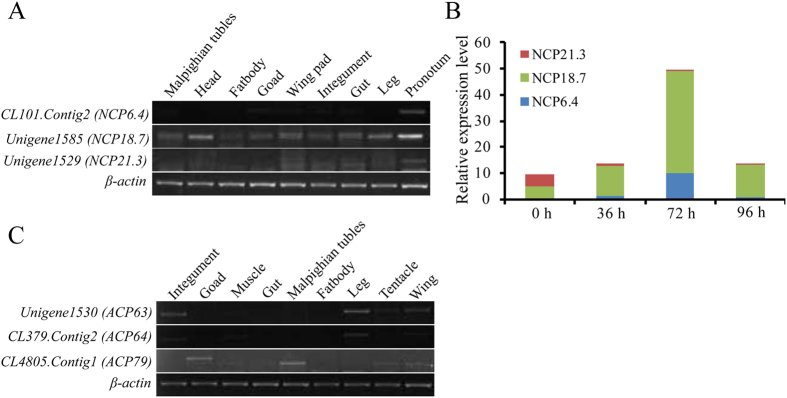
Expression profiles of members of the CPF/CPFL gene families as determined by RT-PCR and RT-qPCR. (**A**) Expression of CPF/CPFL gene-annotated nymph cuticle protein genes in different tissues on day 6 of 5^th^ instar nymphs as detected by RT-PCR (28 cycles). Different tissues are listed: Integument, head, leg, pronotum, goad, gut, Malpighian tubules, fat body, wing pad; (**B**) Expression of CPF/CPFL gene-annotated adult cuticle protein genes in different tissues on day 2 of adults, as detected by RT-PCR (28 cycles). Different tissues are listed: Integument, leg, muscle, tentacle, goad, gut, Malpighian tubules, fat body, wing. *β-actin* was used as the reference control (24 cycles). The image were obtained by using the gel imaging analysis system (Bio-Rad, USA). The full-length gels are presented in Supplementary Fig. 6. (**C**) Expression of CPF/CPFL genes in the pronotum after molt from 4^th^ instar nymphs to 5^th^ instar nymphs at 0 h, 36 h, 72 h and 96 h, as detected by RT-qPCR. *β-actin* was used as the reference control. Data are reported as means ± SE of three independent biological replications.

By searching the *L. migratoria* transcriptome with the CPF/CPFL motif, we obtained nine cuticle protein genes of the CPF/CPFL family. According to CuticleDB, they all were annotated the nymph cuticle protein gene (*NCP*) and adult cuticle protein gene, respectively. We selected six of them to perform tissue expression analysis by RT-PCR. The results indicated that CL101.Contig2 (*NCP6.4*) was only expressed in the pronotum of 5^th^ instar nymphs, whereas Unigene1585 (*NCP18.7*) and Unigene1529 (*NCP21.3*) were expressed in all tested tissues (Fig. 8A). For the adult cuticle protein genes, the expression levels of Unigene1530 (*ACP63*) and CL379.Contig2 (*ACP64*) were similar in the integument, leg, tentacle and wing, while CL4805.Contig1 (*ACP79*) was highly expressed in the gonad, Malpighian tube, tentacle and wing (Fig. 8B). To explore the stage specificity of nymph cuticle protein genes, developmental stage expression after molting from 4^th^ instar nymphs revealed that the expression of CL101.Contig2 (*NCP6.4*) and Unigene1585 (*NCP18.7*) were gradually increased and exhibited a peak level at 72 h, followed by a decrease at 96 h after the molting of 4^th^ instar nymphs, while Unigene1529 (*NCP21.3*) had the highest expression level at 0 h after molting and then gradually decreased (Fig. 8C). Thus, all of these genes have a stage specificity in the development and molting of *L. migratoria*, and their function might be different.

## Discussion

In the present study, several criteria were used to identify a cuticular protein gene: (1) a known cuticular protein consensus, such as R&R Consensus and 44 amino-acid residues, (2) a simple repeat sequence (AAP(A/V)), and (3) sequence similarity to known cuticular proteins. Finally, we identified 81 putative cuticular protein genes in *L. migratoria* transcriptome database (Table 1). Among these genes, 7 were falsely annotated. We then revised these gene structures and named the genes (Table S4). The CPR family is the most abundant family of cuticular proteins in *L. migratoria* and comprises 51 R&R proteins (63%), which is fewer than the numbers found in *D. melanogaster* (101 R&R proteins)^8^, *B. mori* (148 R&R proteins)^20^ and *Anopheles gambiae* (156 R&R proteins)^31^, but more abundant than that in *A. mellifera* (28 R&R proteins)^19^.

In *T. castaneum*, 17 genes encoding two families of CPAPs were evaluated. According to the chitin-binding domain (ChtBD2), they have been divided into two families, CPAP1 and CPAP3, which containing one and three ChtBD2, respectively. These genes were expressed specially in cuticle-forming tissues^9^. In *L. migratoria*, we obtained 9 CPAP gene sequences by searching the transcriptome database that is less than *T. castaneum*, including 2 CPAP1 and 7 CPAP3 containing one and three ChtBD2, respectively (Fig. 6A). Evolutionary tree analysis showed that they form a cluster with *D. melanogaster* and *T. castaneum*, respectively (Fig. 6B).

We also found 2 cuticular proteins with the Tweedle motif, which form a cluster with the Tweedle proteins of *A. mellifera* and *Nasonia vitripennis*, respectively (Fig. 7A). The exception was the Tweedle protein, which was in contrast to *D. melanogaster*, where Tweedle proteins form large clusters^12^. In addition, these proteins contain a series of a low complexity of short repetitive sequences in the C-terminus, such as GGGI, GGGL, GGGSI, and GGGSL, similar to that of *B. mori*. It is worth noting that these repeat short sequences might be related to the structure and properties of the cuticle proteins that contain the Tweedle motif by forming a hydrophobic area.

In contrast to *D. melanogaster* and *A. gambiae*, which have three or four CPF genes^16^, 9 CPF/CPFL proteins were found from the *L. migratoria* transcriptome (Table S4). These cuticle proteins contain many short tandem repeats, including AAH, AAPA/V, and AAAPL. It was reported that these motifs repeated in the cuticle proteins, and usually play a role in the formation of a tertiary structure to change the orientation of a polypeptide chain at the P site of AAPA/V by forming a helix-turn^2^,^32^,^33^. Although they have such a conserved motif, it has been proven that the CPF/CPFL proteins do not bind to chitin in *A. gambiae*^16^. Thus, it needs further experimental evidence to confirm its function in *L. migratoria*. We also found 10 other genes with no conserved motif, and their function is unclear.

Many researchers previously thought that each cuticular protein gene within the same cluster should have a similar expression patterns. However, our results showed that adjacent cuticular protein genes have a different spatial and temporal expression within the same cluster (Figs 5, 6, 7, 8), a finding similar to that of *B. mori*^20^. One possibility is that the genes in the clusters have been rearranged or varied from the structure and regulation during evolution. According to the results of Cox and Willis (1985), the composition of cuticular proteins was related to the flexibility of the mature cuticle^34^. Our results showed that the RR-1 protein genes we tested were highly expressed in the integument and pronotum, and some of them showed specificity in the pronotum (Fig. 5A). The expression pattern of these genes is consistent with the synthesis of cuticle (exocuticle and endocuticle), which suggests that they might be involved in the synthesis of cuticle. For RR-2 protein genes as a test, they were all highly expressed in cuticle-forming tissues such as the wings, integument and leg. Among them, *ACP7* and *ACP8* were specifically expressed in the wings at the transcript level (Fig. 5C), which is consistent with previous studies^35^,^36^, a finding that is similar to that in *B. mori* with tissue and stage-specific expression in the wing disc^20^. In contrast to RR-1 and RR-2 proteins, RR-3 proteins are small in number and have been poorly understood. These genes did not display tissue specificity (Fig. 5F), but they were all gradually increased after molting, were at the highest level at 72 h, and then were gradually decreased (Fig. 5H), which is consistent with the synthesis of endocuticle. Our results suggest that RR-3 proteins might function in specific cuticle structure. Meanwhile, we also found several cuticle protein genes are not specific, which more than one bands could be detected, such as NCP21.3, ACP20, Tweedle1, and ACP79. We speculate that it may be different alternative splicing forms of these genes. For example, ACP79 gene have two alternative splicing forms as reported in cuticleDB, LmACP79a and LmACP79b, but we only found one form in this transcriptme since they are different in only partly acid amino. In addition, Tweedle gene was found two forms in *L. migratoria*, but four forms in *B. mori*. Through a BLASTP against the nr database in NCBI, NCP21.3 and ACP20 have two isoforms in *Cephus cinctus* and *Cyphomyrmex costatus, Stomoxys calcitrans* and *Rhagoletis zephyria*, respectively. Whether there is any other forms in *L. migratoria* need to further study.

Functions of CPAP genes in *T. castaneum* were analyzed by RNAi assay, it was found that most CPAPs play an essential role in the cuticle formation of different tissues and developmental stages as tested^9^. In *D. melanogaster*, similar results of the non-redundancy of CPAP functions have also been obtained in exoskeleton organization and tracheal tubulogenesis^37^,^38^. These results not only reveal specialized functions of CPAP proteins, but also provide a referable value for the CPAP proteins conserved in different insect species. Furthermore, it is the first time to conduct comprehensively functional analysis for the CPAP family proteins in any insect species^9^. In *L. migratoria*, CPAP genes have a different expression pattern, although they cluster the same family (Fig. 6). Thus, further study of its functions is needed in the formation of cuticle in *L. migratoria*.

In *L. migratoria*, Tweedle protein genes were expressed in almost all of the test tissues, which is different from that of *D. melanogaster* and *B. mori*, where it is mainly expressed in the epidermis and wing disc^12^,^20^. Additionally, their expression in different stages is distinct (Fig. 7C), suggesting they may have a different function in the formation of cuticle structures. Other than *Tweedle* genes, some CPF and CPFL genes have tissue and stage-specific expression in *L. migratoria* (Fig. 8). Similarly, Togawa *et al*.^16^ found that four CPFs and one CPFL protein genes had a high expression level just before pupation or adult emergence in *A. gambiae* and other insects, suggesting that these proteins are correlated with the formation of outer layer of pupal or adult cuticles (epicuticle and exocuticle). However, six CPFL protein genes mainly expressed before larval/larval molts which is different from that of CPF protein genes and may be related to the synthesis of larval cuticles^16^. According to our results, CPFs and CPFLs proteins may have relation to the formation of cuticle in the integument and wing pads, which is similar to RR-2 proteins and Tweedle proteins. However, there are still some cuticular protein genes with unexpected expression in the tissues not derived from ectoderm such as the goad, Malpighian tubule, and fat body (Figs 5, 6, 7, 8). Similar results were found in other species, such as *B. mori*^20^ and *Manduca sexta*^39^. It has been speculated that these proteins are formed and transported to the cuticle-forming tissues from the internal tissues, or its transcripts of these protein genes originated in the attached trachea that possess the epidermic structure^6^.

In *B. mori*, two expression patterns of pupal cuticular protein genes have been previously described. They were transcribed after the beginning of wandering and after the pre-pupa stage when ecdysteroid titer is very high, respectively^20^,^40^. The insect cuticle is composed of several different layers such as the envelope, epicuticle and procuticle, which are synthesized under the regulation of different hormonal. Epicuticle and procuticle have a great distinction in hormonal regulation^41^,^42^. In the present study, several cuticlar protein genes were identified and shown the stage-specific in wing pads (Fig.5), which is in accordance with ecdysteroid titer, suggesting that the expression of these genes may be regulated by ecdysone signal.

## Materials and Methods

### Experimental animal and RNA isolation

The migratory locusts *L. migratoria* were reared with fresh wheat seedlings and wheat bran at 28–30 °C and 60% relative humidity with 14:10 h light: dark cycle in the laboratory. The whole body of *L. migratoria* at different developmental stages including egg, 1, 2^nd^, 3^rd^, 4^th^, 5^th^ instar nymph and adult were collected and combined, and total RNA was isolated using TRizol Reagent (TaKaRa, Japan) according to the manufacturer’s protocol. Total RNA was dissolved in H_2_O and RNA purity was checked using a Nanodrop ND-2000 spectrophotometer (Thermo, USA), and RNA integrity was assessed using an Agilent 2100 BioAnalyzer (Agilent Technologies, California, USA).

### Library construction and Illumina sequencing

Poly(A) mRNAs were isolated and enriched from 10 μg of total RNA using oligo (dT) magnetic beads. Purified mRNAs were fragmented (200 nt to 700 nt) with RNA Fragmentation Reagent and reverse transcribed into cDNA using Super Script II Reverse Transcriptase (Invitrogen, USA) following the manufacturer’s protocols, followed by second-strand cDNA synthesis in reaction mixtures containing 1 × buffer, dNTPs, RNase H, and DNA polymerase I. The resulting double-stranded cDNA (dsDNA) was then purified using the Agencourt^®^ AMPure^®^ XP beads (Beckman Coulter Inc., Beverly, MA, USA) and resolved with EB buffer for end reparation and adding poly(A). After that, fragments were then ligated to sequencing adapters, and enriched by PCR amplification to obtain adequate fragments for the final cDNA library. Amplified products were purified with QiaQuick Gel Extraction Kit (QiaGen, Germany), and the library was sequenced using the Illumina HiSeq 2000 platform (Illumina, San Diego, CA, USA) at the Beijing Genomics Institute (BGI, Shenzhen, China).

### Assembly and annotation of transcriptomes

The raw data outputs from the Illumina equipment were trimmed for adapters and polyA/T tails and low-quality reads (Q20 less than 20) were removed to obtain high-quality, clean reads. The clean reads were assembled to produce Unigenes with the Trinity short read assembly program^43^. For functional annotations, the assembled Unigenes were aligned with the nr, nt, SwissProt, COG, and KEGG databases using BLAST with a cut-off E-value of 10^−5 ^44. The coding region sequences (CDS) were extracted from the Unigene sequences based on the BLAST results, and translated into peptide sequences. In addition, a Unigene without homology to these databases was predicted for the direction of the sequence using the ESTScan software^45^. Besides, the Gene Ontology (GO) annotation of all Unigene sequences was collected and analyzed using Blast2GO program (http://www.blast2go.org) according to the GO association done by a BLASTX against the nr database^46^,^47^.

### Identification and sequence analysis of cuticle protein genes from *L. migratoria*

First, we identified genes that might code for cuticular proteins with the following known motif: for the CPR family, the R&R Consensus^13^,^48^, containing RR1, RR2 and RR3 were used; classification into the appropriate subfamily was confirmed using a profile hidden Markov model that discriminates between the two subtypes, available at the cuticleDB website^8^. For *Tweedle* genes, the Tweedle motif was used to identify homologs^12^. For the CPAP family, the *Tribolium castaneum* CPAP1 and CPAP3 sequences from NCBI were used to identify CPAP family members. CPF and CPFL genes were identified using the most highly conserved portion of the 44 amino-acid motif, VSxYSKAVDTPFSSVRKxDxRIVNxA and LxYSAAPAVSHVAYxGxGxxYGW, respectively^16^. In addition to these known motifs, putative cuticular protein genes were predicted using the simple repeat sequence (GGX) and sequence similarity to known cuticular proteins^20^. The potential candidates of *L. migratoria* cuticle protein genes were further confirmed via screening the BLASTX search algorithm against the NCBI nr database with a cut-off E-value of 10^−5^.

The amino acid sequences of putative cuticle protein genes identified above were predicted by Compute pI/Mw tool (http://www.expasy.org/compute_pi/) for the molecular mass (MM) and isoelectric point (pI). The domain architecture and signal peptide of them were analyzed by SMART domain analysis (http://smart.emblheidelberg.de/) and SignalP 4.1 Server (http://www.cbs.dtu.dk/services/SignalP/). The Weblogo online server was used to identify the conserved elements in CPR protein genes (http://weblogo.berkeley.edu/logo.cgi).

### Phylogenetic analysis

The amino acid sequences of cuticular protein genes of *D. melanogaster, A. mellifera, B. mori* and *T. castaneum* were obtained from the cuticleDB website or NCBI, as described above. These sequences were used for sequence comparisons and constructing phylogenetic trees by the neighbor-joining method using MEGA5 software^49^. The GenBank accession numbers are listed in Table S1.

### Expression analysis of several identified cuticle protein genes

To determine the expression patterns of several key cuticle protein genes in nymphs and adults, we first dissected the different tissues of fifth instar nymphs as described above including the integument, gut, Malpighian tubules, fat body, wing pads, goads and pronotum, and extracted total RNA for the expression analysis of nymph cuticle protein genes. We dissected the different tissues of adults for expression analysis of adult cuticle protein genes, including the integument, gut, Malpighian tubules, fat body, wing, goads, muscle, tentacle and leg. For expression analysis of several key cuticle protein genes at different development stages, we dissected and extracted total RNA of the whole body, wing pad or wing from the embryo, nymph to adult; and wing pad (day 1, day 3, day 5) from 4^th^ instar nymphs; integument (day 1, day 3, day 5, day 7) from 5^th^ instar nymphs; and integument or pronotum in molting (0 h), 36 h, 72 h and 96 h after molting from 4^th^ instar nymphs to 5^th^ instar nymphs, respectively. One μg of total RNA was used to synthesize first-strand cDNA by using M-MLV reverse transcriptase (TaKaRa, Japan). Each cDNA sample was diluted 20-fold for use as a template.

For reverse-transcription PCR (RT-PCR), the initial denaturation was at 94 °C for 5 minutes, annealing at (55–60 °C) for 30 s and the final extension was at 72 °C for 10 minutes using gene-specific primers. RT-PCR products for the *L. migratoria β-actin* gene from the same cDNA templates served as an internal control for loading (24 cycles). The PCR products were subjected to electrophoresis and the results were analyzed by gel imaging and analysis system (SYSTEM GelDoc XR, Bio-Rad, USA). For reverse-transcription quantitative PCR (RT-qPCR) analysis and SYBR Green kits were used according to the manufacturer’s instructions (TaKaRa, Japan) with specific primers for each gene designed and listed in Table S2. The total volume of RT-qPCR reactions was 20 μl, containing 10 μl of 2 × SYBR^®^ Premix EX Taq™ (TaKaRa, Japan), 0.4 μl of 50 × ROX Reference Dye (TaKaRa, Japan) and 0.4 μl of specific primers (10 μM), with the following conditions: denaturation at 95 °C for 1 min, followed by 40 cycles at 95 °C for 15 s, 60 °C for 31 s with an ABI 7300 real-time PCR machine (Applied Biosystems, USA) using FastStart Universal SYBR Green Master. A melting curve was determined for each sample to detect the gene-specific peak and check for the absence of primer-dimers. The relative mRNA levels of target genes were calculated using the 2^−ΔΔCt^ method^50^, and the target gene expression level was normalized to the expression of the internal marker gene *β-actin*^51^. Three independent biological replicates were performed. All of the data were statistically analyzed by independent sample student *t-test*.

## Additional Information

**How to cite this article:** Zhao, X. *et al*. Identification and expression of cuticular protein genes based on *Locusta migratoria* transcriptome. *Sci. Rep.* **7**, 45462; doi: 10.1038/srep45462 (2017).

**Publisher's note:** Springer Nature remains neutral with regard to jurisdictional claims in published maps and institutional affiliations.

## Supplementary Material

Supplementary Information

## Figures and Tables

**Table 1:** Numbers of cuticle protein genes from *L. migratoria* transcriptome.

Family	Number of genes
CPR (RR-1)	25
CPR (RR-2)	18
CPR (RR-3)	8
CPAPs	9
Tweedle	2
CPF/CPFLs	9
Others	10
Total	81
