# Supplementary material for: Identification and expression of cuticular protein genes based on Locusta migratoria transcriptome
Source: Sci Rep. 2017 Apr 3;7:45462. doi: 10.1038/srep45462 (PMC5377371; doi:10.1038/srep45462)
Supplement: Supplementary Information [file srep45462-s1.pdf]

Identification and expression of cuticular protein genes based on *Locusta migratoria* transcriptome

Xiaoming Zhao[1#], Xin Gou[1,2#], Zhongyu Qin[1,2], Daqi Li[1], Yan Wang[1,2], Enbo Ma[1], Sheng Li[3] and Jianzhen Zhang[1] *

[1]Research Institute of Applied Biology, Shanxi University, Taiyuan, Shanxi 030006, China

[2]College of Life Science, Shanxi University, Taiyuan, Shanxi 030006, China

[3]Guangzhou Key Laboratory of Insect Development Regulation and Application Research, Institute of Insect Sciences and School of Life Sciences, South China Normal University, Guangzhou 510631, China

Supplementary legends

Figure S1 Output statistics of sequencing, statistics of assembly quality and length distribution of Unigene

Figure S2. Gene ontology (GO) assignment for the *L. migratoria* transcriptome. The GO classification map was done by uploading the GO ID numbers of genes for their involvement in biological processes, cellular components, and molecular functions. The number of Unigenes assigned to each GO term is shown in the right column.

Figure S3. Clusters of orthologous groups (COG) classification of *L. migratoria* Unigenes. A total of 9,160 produced functional annotations were among the 25 categories. The Y-axis shows the number of unigene in each COG term.

Figure S1

A

Output statistics of sequencing

Samples	Total Raw Reads	Total Clean Reads	Total Clean Nucleotides (nt)	Q20 percentage	N percentage	GC percentage
N2	59,129,922	53,559,770	4,820,379,300	97.42%	0.00%	46.44%

B

Statistics of assembly quality

	Sample	Total Number	Total Length(nt)	Mean Length(nt)	N50	Total Consensus Sequences	Distinct Clusters	Distinct Singletons
Contig	N2	188,554	49,092,957	260	352	-	-	-
Unigene	N2	84,641	41,542,996	491	692	84,641	13,352	71,289

C

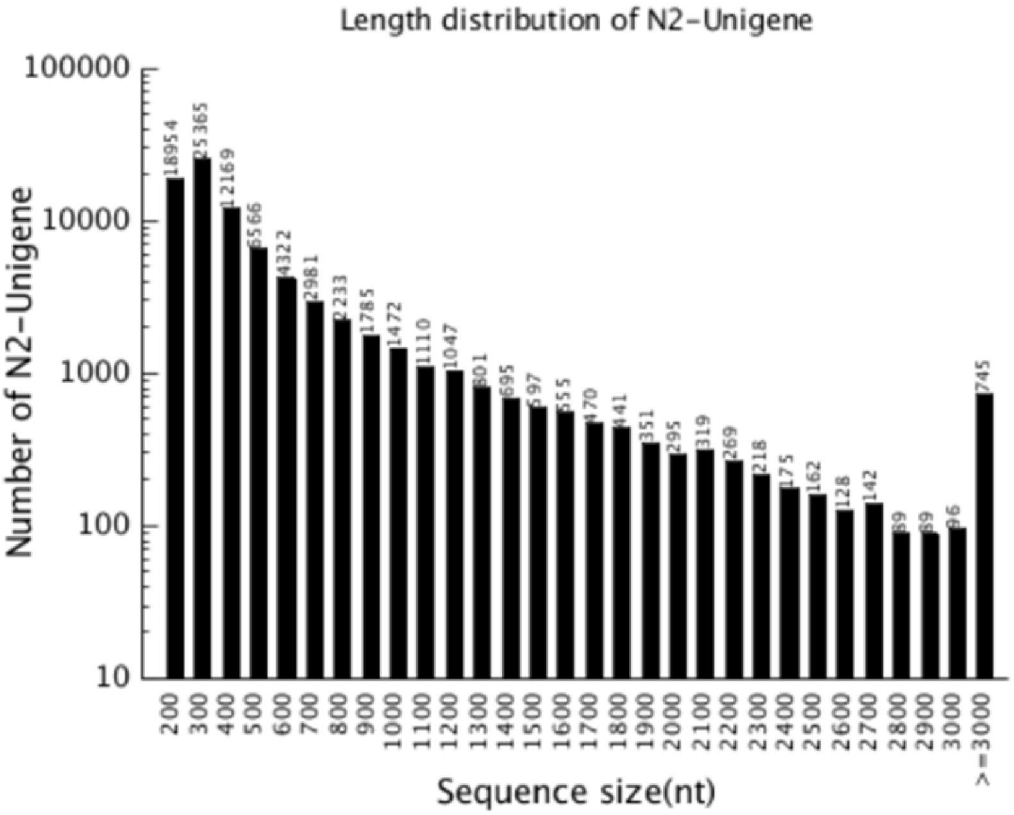

Figure S2

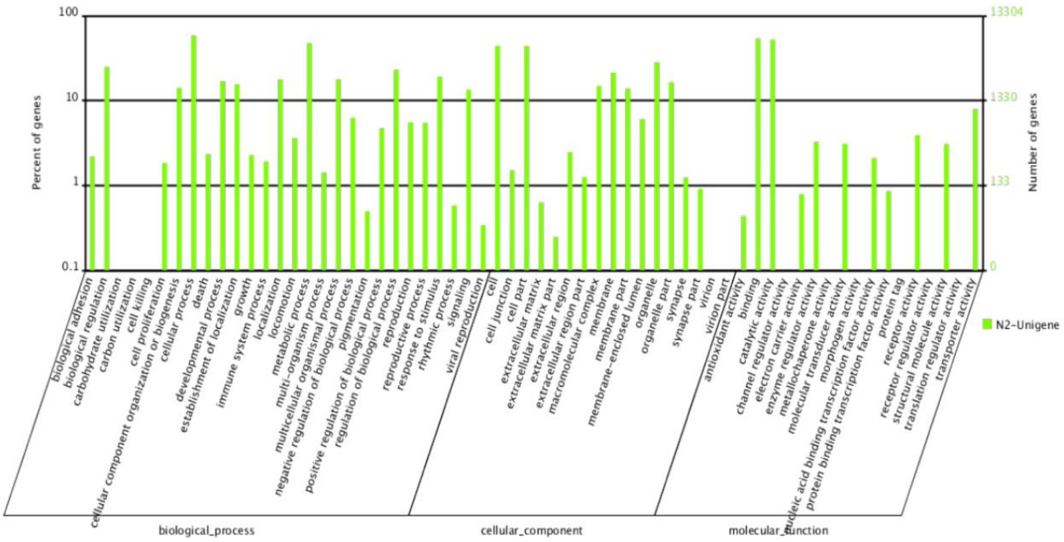
100
10
1
0.1
Percent of genes
13304
1330
133
0
Number of genes
biological adhesion
biological regulation
carbohydrate utilization
carbon utilization
cell killing
cell proliferation
cellular component organization or biogenesis
cellular process
death
developmental process
establishment of localization
growth
immune system process
localization
locomotion
metabolic process
multi-organism process
multicellular organismal process
negative regulation of biological process
pigmentation
positive regulation of biological process
regulation of biological process
reproduction
reproductive process
response to stimulus
rhythmic process
signaling
viral reproduction
cell
cell junction
cell part
extracellular matrix
extracellular matrix part
extracellular region
extracellular region part
macromolecular complex
membrane
membrane part
membrane-enclosed lumen
organelle
organelle part
synapse
synapse part
virion
virion part
antioxidant activity
binding
catalytic activity
channel regulator activity
electron carrier activity
enzyme regulator activity
metallochaperone activity
molecular transducer activity
morphogen activity
nucleic acid binding transcription factor activity
protein binding transcription factor activity
protein tag
receptor activity
receptor regulator activity
structural molecule activity
translation regulator activity
transporter activity
N2-Unigene
biological_process
cellular_component
molecular_function

Figure S3

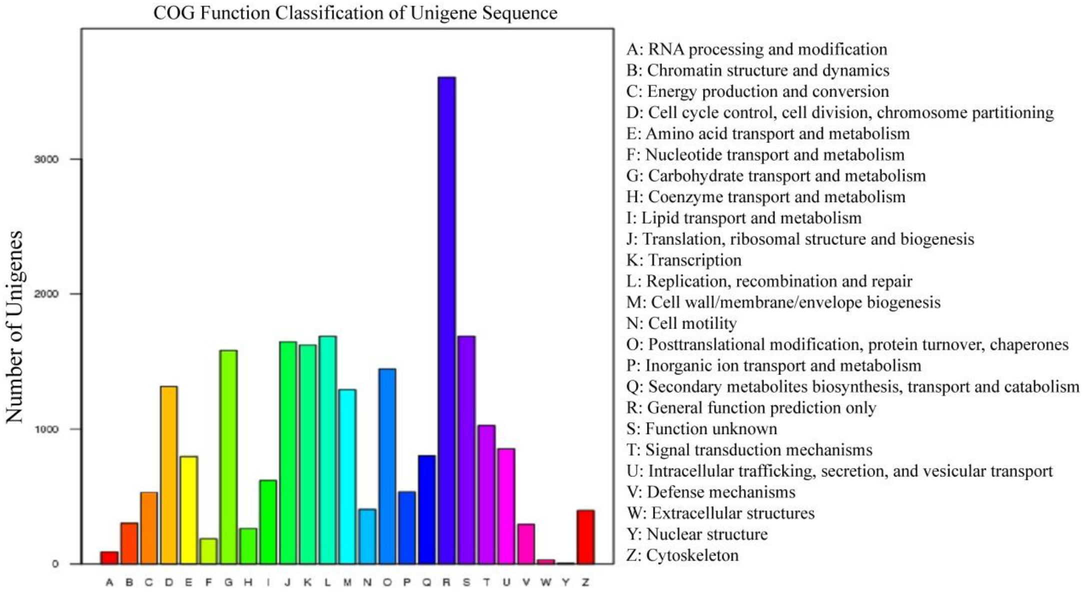
COG Function Classification of Unigene Sequence
Number of Unigenes
3000
2000
1000
0
A B C D E F G H I J K L M N O P Q R S T U V W Y Z
A: RNA processing and modification
B: Chromatin structure and dynamics
C: Energy production and conversion
D: Cell cycle control, cell division, chromosome partitioning
E: Amino acid transport and metabolism
F: Nucleotide transport and metabolism
G: Carbohydrate transport and metabolism
H: Coenzyme transport and metabolism
I: Lipid transport and metabolism
J: Translation, ribosomal structure and biogenesis
K: Transcription
L: Replication, recombination and repair
M: Cell wall/membrane/envelope biogenesis
N: Cell motility
O: Posttranslational modification, protein turnover, chaperones
P: Inorganic ion transport and metabolism
Q: Secondary metabolites biosynthesis, transport and catabolism
R: General function prediction only
S: Function unknown
T: Signal transduction mechanisms
U: Intracellular trafficking, secretion, and vesicular transport
V: Defense mechanisms
W: Extracellular structures
Y: Nuclear structure
Z: Cytoskeleton

Fig.S4-1

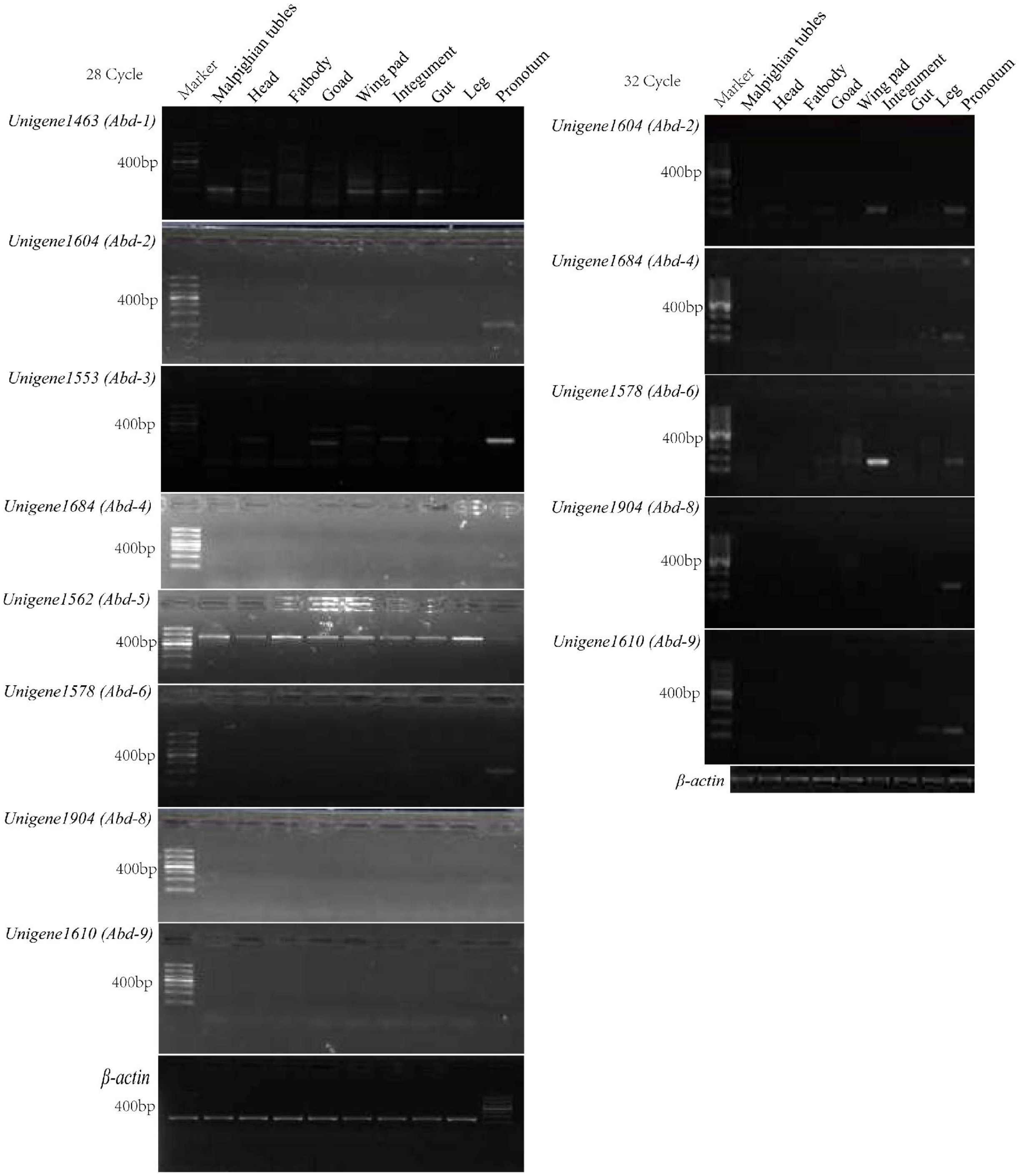
28 Cycle
Marker
Malpighian tubles
Head
Fatbody
Goad
Wing pad
Integument
Gut
Leg
Pronotum
Unigene1463 (Abd-1)
400bp
Unigene1604 (Abd-2)
400bp
Unigene1553 (Abd-3)
400bp
Unigene1684 (Abd-4)
400bp
Unigene1562 (Abd-5)
400bp
Unigene1578 (Abd-6)
400bp
Unigene1904 (Abd-8)
400bp
Unigene1610 (Abd-9)
400bp
β-actin
400bp
32 Cycle
Marker
Malpighian tubles
Head
Fatbody
Goad
Wing pad
Integument
Gut
Leg
Pronotum
Unigene1604 (Abd-2)
400bp
Unigene1684 (Abd-4)
400bp
Unigene1578 (Abd-6)
400bp
Unigene1904 (Abd-8)
400bp
Unigene1610 (Abd-9)
400bp
β-actin

Fig.S4-2

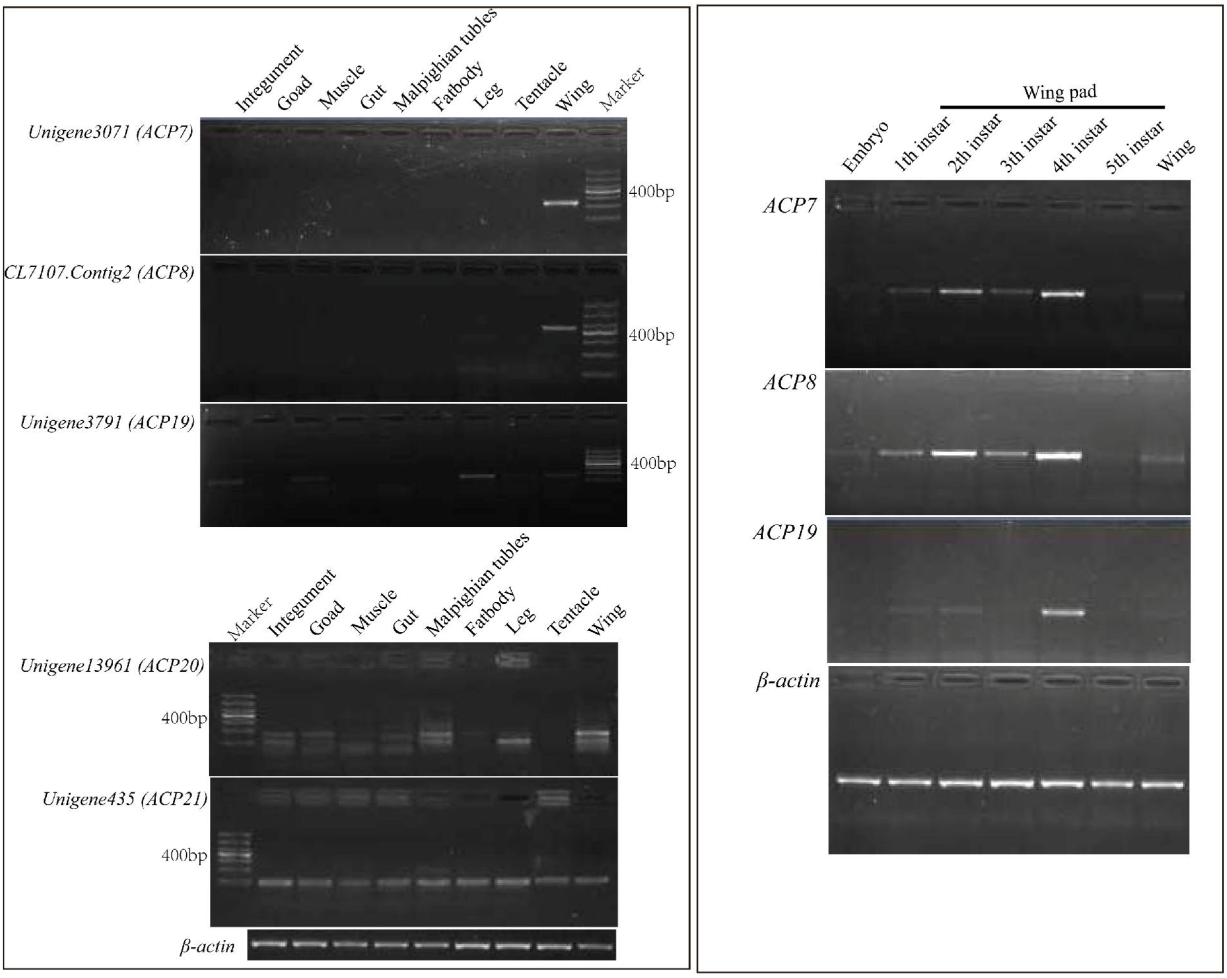
Integument
Goad
Muscle
Gut
Malpighian tubles
Fatbody
Leg
Tentacle
Wing
Marker
Unigene3071 (ACP7)
400bp
CL7107.Contig2 (ACP8)
400bp
Unigene3791 (ACP19)
400bp
Marker
Integument
Goad
Muscle
Gut
Malpighian tubles
Fatbody
Leg
Tentacle
Wing
Unigene13961 (ACP20)
400bp
Unigene435 (ACP21)
400bp
β-actin
Wing pad
Embryo
1th instar
2th instar
3th instar
4th instar
5th instar
Wing
ACP7
ACP8
ACP19
β-actin

Fig.S4-3

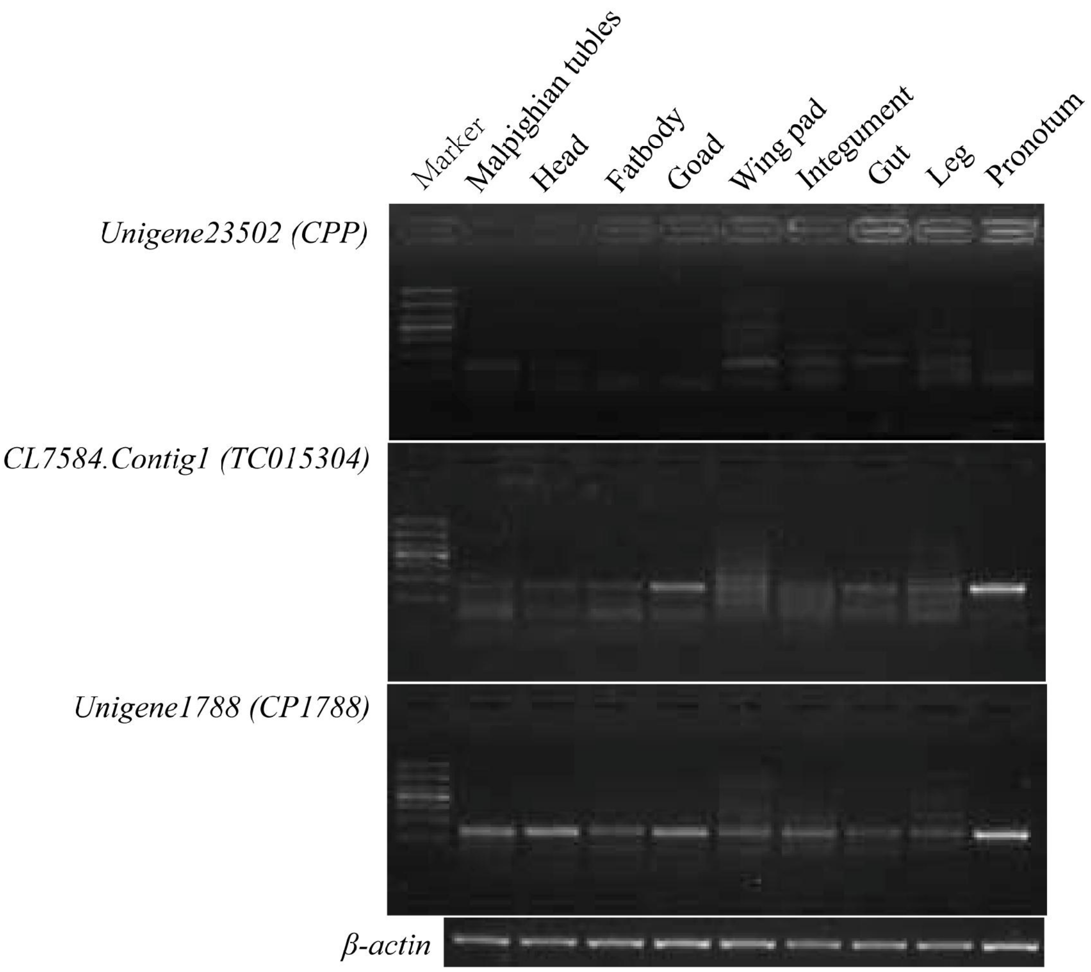
Marker
Malpighian tubles
Head
Fatbody
Goad
Wing pad
Integument
Gut
Leg
Pronotum
Unigene23502 (CPP)
CL7584.Contig1 (TC015304)
Unigene1788 (CP1788)
β-actin

Fig.S5

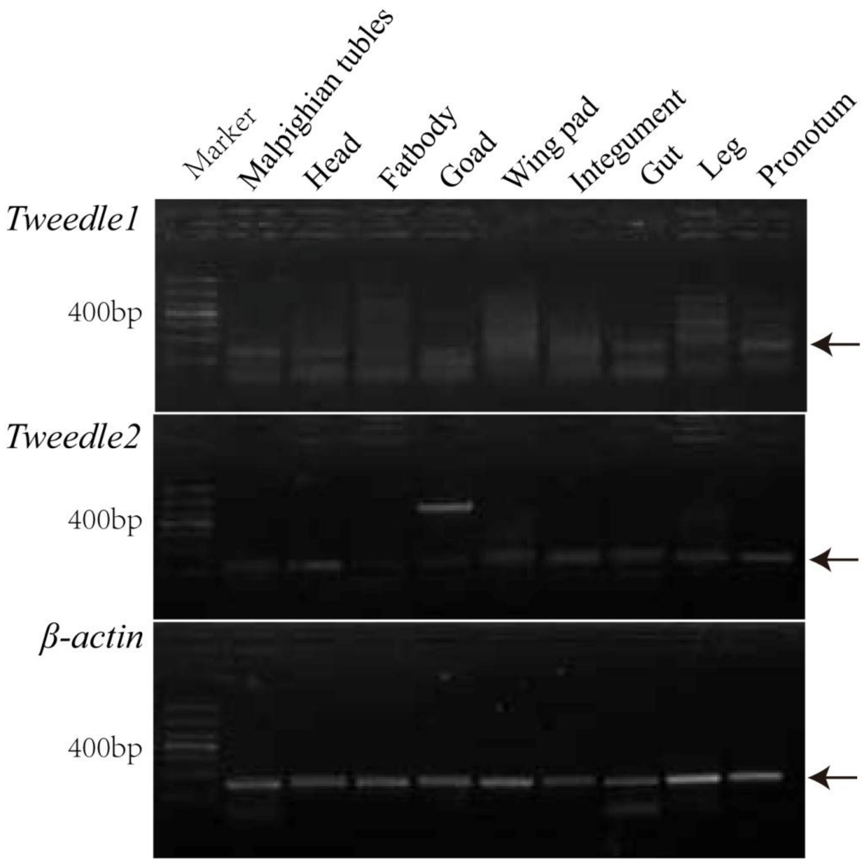
Marker
Malpighian tubles
Head
Fatbody
Goad
Wing pad
Integument
Gut
Leg
Pronotum
Tweedle1
400bp
Tweedle2
400bp
β-actin
400bp

Fig.S6

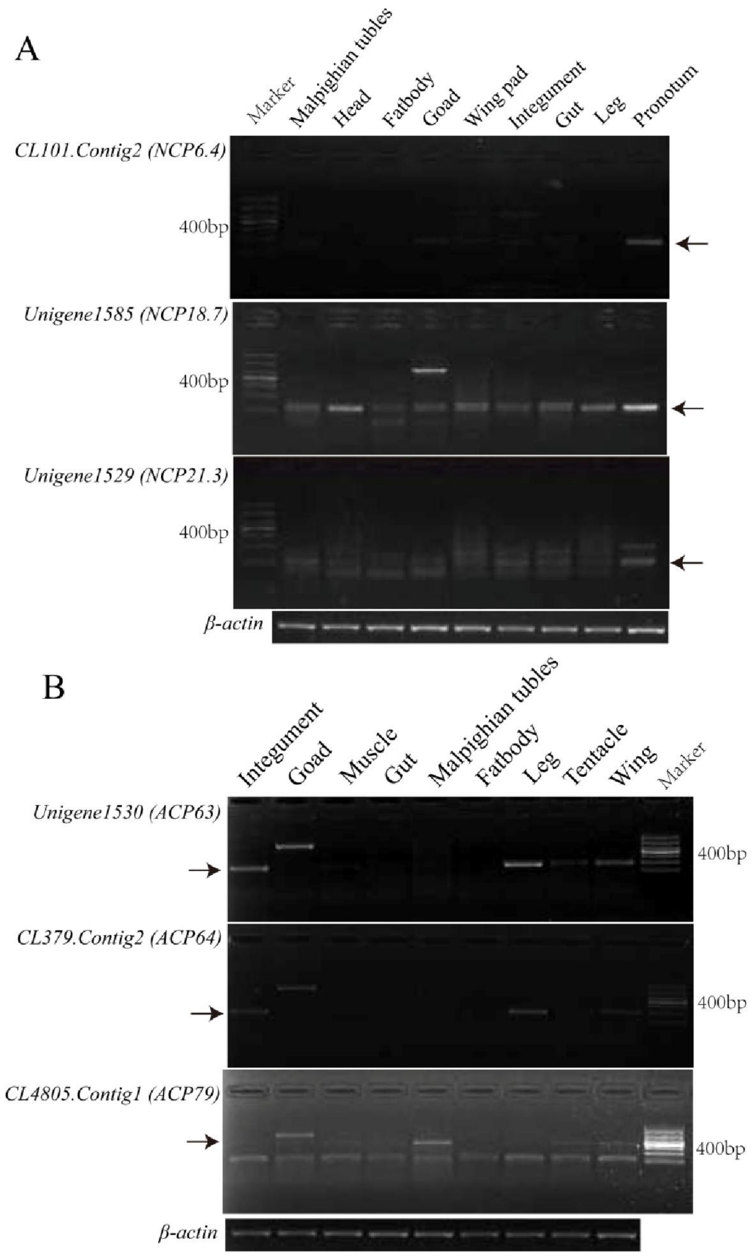
A
Marker
Malpighian tubles
Head
Fatbody
Goad
Wing pad
Integument
Gut
Leg
Pronotum
CL101.Contig2 (NCP6.4)
400bp
Unigene1585 (NCP18.7)
400bp
Unigene1529 (NCP21.3)
400bp
β-actin
B
Integument
Goad
Muscle
Gut
Malpighian tubles
Fatbody
Leg
Tentacle
Wing
Marker
Unigene1530 (ACP63)
400bp
CL379.Contig2 (ACP64)
400bp
CL4805.Contig1 (ACP79)
400bp
β-actin

Table S1: Name of gene and accession number for phylogenetic analysis from NCBI

Homologous gene	Insects	Accession number
LmObst-A1	*Locusta migratoria*	KM099158
LmObst-A2	*Locusta migratoria*	KM099161
LmObst-B	*Locusta migratoria*	KM099159
LmObst-C	*Locusta migratoria*	KM099160
LmObst-D1	*Locusta migratoria*	KM099162
LmObst-D2	*Locusta migratoria*	KM099163
LmObst-E	*Locusta migratoria*	KM099164
LmObst-F	*Locusta migratoria*	KX503043
LmObst-H	*Locusta migratoria*	KX503044
DmObst-A1	*Drosophila melanogaster*	NP_608378.2
TcObst-A1	*Tribolium castaneum*	NP_001161910.1
TcObst-A2	*Tribolium castaneum*	NP_001161909.1
DmObst-B	*Drosophila melanogaster*	NP_609339.1
TcObst-B	*Tribolium castaneum*	NP_001073566.1
DmObst-C1	*Drosophila melanogaster*	NP_649611.2
DmObst-C2	*Drosophila melanogaster*	NP_001097694.1
TcObst-C1	*Tribolium castaneum*	NP_001073569.1
TcObst-C2	*Tribolium castaneum*	NP_001107778.1
DmObst-D	*Drosophila melanogaster*	NP_523418.1
TcObst-D1	*Tribolium castaneum*	NP_001161908.1
TcObst-D2	*Tribolium castaneum*	NP_001073568.1
TcObst-E	*Tribolium castaneum*	NP_001161915.1
DmObst-E1	*Drosophila melanogaster*	NP_608957.1
DmObst-E2	*Drosophila melanogaster*	NP_723116.1
TcObst-F	*Tribolium castaneum*	NP_001161917.1
TcObst-H	*Tribolium castaneum*	NP_001161918.1
Tweedle1	*Apis mellifera*	ACJ38118
Tweedle1	*Nasonia vitripennis*	NP_001136080
Tweedle2	*Apis mellifera*	ADK73965
Tweedle2	*Nasonia vitripennis*	NP_001166856
Tweedle2	*Bombyx mori*	NP_001036950
Tweedle3	*Bombyx mori*	NP_001036951
Tweedle4	*Bombyx mori*	NP_001166627

Table S2 Primer sequences used in this study

Gene	Primer sequences (5′-3′)	Assay
Abd1	F: TCCAGCGGTAGGAGCCGTCGTT<br>R: AGCCCGCCATCGTGCCAGTT	RT-PCR and RT-qPCR
Abd2	F: GTGTTTGACCCTGCTGGTG<br>R: CCGTTGCCGTTTCGTATT	RT-PCR and RT-qPCR
Abd3	F: CCGTTCTCGTCGGCGGTGTA<br>R: ACGTCAACTTCGATGGCTCCT	RT-PCR and RT-qPCR
Abd4	F: CGCACTTCCCGACTCCAC<br>R: CGGGGCTCTGCCTTATCG	RT-PCR and RT-qPCR
Abd5	F: AGTCCTAAGCGCCCACAATG<br>R: GGCAAATAACTTCACAAAAT	RT-PCR and RT-qPCR
Abd6	F: GACGGCAAGGACTACAGCA<br>R: TCGAGTGAGGAGGGAGGAA	RT-PCR and RT-qPCR
Abd8	F: AGCAGCCGGGCAACAACC<br>R: CGTCACATCAGCAACAGCGAAG	RT-PCR and RT-qPCR
Abd9	F: AGGACCTCAGCACCCCAACC<br>R: CGGGACAAGCCCAAAAGAGT	RT-PCR and RT-qPCR
ACP7	F: CAGTCAGCTGCGTCCACCTC<br>R: GGCGCGCGCGACGGCCACCA	RT-PCR and RT-qPCR
ACP8	F: GAAGGTACGGCAACCTGGGA<br>R: GGGGGCGGCCACGTGGGCGA	RT-qPCR
ACP8	F: GGGGGCTACGCCGCCCCCGC<br>R: GTGGTAGGCGATGGGCGCATG	RT-PCR
ACP19	F: TCCACCGACGAGCAGACCAG<br>R: TTGACGACGGCGGGAGCAGC	RT-PCR and RT-qPCR
ACP20	F: CGTATGGCGTGACGGACC<br>R: GCTGACGACGGCGTTGAA	RT-PCR and RT-qPCR
ACP21	F: GCCGTGGCTGCCGAGTAT<br>R: GGTCTCCTGCTGGGTCTTG	RT-PCR and RT-qPCR
cpp	F: GGGCAGCTATCGGCCAAGCA<br>R: ACGCGGAAGCCGTTCAGGTC	RT-PCR and RT-qPCR
TC014498	F: CAAGGCTCTGAGGACGAAG<br>R: TCACCCTGTGGCTGGAAT	RT-PCR and RT-qPCR
CP1788	F: GTCGGCGGCTACTCCTACGT<br>R: TGGATGCGGTGGATGACTT	RT-PCR and RT-qPCR
LmObst-A1	F: GCTGCTCAGTCGGAGAGGTCTA<br>R: AGTCCTCACATCCTGGCACG	RT-qPCR
LmObst-A2	F: GTCACCTTCAAGTGCGACGA<br>R: CCTCTTGCCTTCATCAGCGT	RT-qPCR
LmObst-B	F: AGGAACTACTCGCTCCCACG<br>R:GAGGCGAGTAAGTCTGTAAAACGT	RT-qPCR
LmObst-C	F: ATCGGAGACTCCGACGGCT<br>R: TCGCTCTTGCGGATGCTCT	RT-qPCR

LmObst-D1	F: GAAATAAGCCAACCATCTGC<br>R:CTGTCAGGACTTTACATACACAATT	RT-qPCR
LmObst-D2	F: CTGGGCAAGTCGTGCAACC<br>R: GACGGTCGAACACCAGCATG	RT-qPCR
LmObst-E	F: GAGATGTGCTGATGGCTACGC<br>R: GCAGGCTTCAGTCGGAGATTAG	RT-qPCR
LmObst-F	F: AGTATGGCACTGGTGTCTC<br>R: TTCCGTCTTTGTCCTTGTAT	RT-qPCR
LmObst-H	F: TATCATCATTGCCTCTTTG<br>R: ACAGGGCTTCATTTCTTTT	RT-qPCR
Tweedle1	F: TTCATCCGCTACAAGACCCAGA<br>R: CCTCCGAGACCGTCACCG	RT-PCR and RT-qPCR
Tweedle2	F: CGTGGTGCCCAGCGTGTA<br>R: ACGGCAGGGGTGTCCAGT	RT-PCR and RT-qPCR
NCP6.4	F: GGATACTATGGCTACGGC<br>R: CGATTATGTTTACAGAGGC	RT-PCR and RT-qPCR
NCP18.7	F: CGTGGTGCCCAGCGTGTA<br>R: ACGGCAGGGGTGTCCAGT	RT-PCR and RT-qPCR
NCP21.3	F: TACACCAACACCGCCGACTT<br>R: CACCGCCTTGGAGAACTGC	RT-PCR and RT-qPCR
ACP63	F: ACGGACACGGTCTGGGATA<br>R: CGAGGGTGAAGCGTAGTTT	RT-PCR
ACP64	F: TCAGTGATGGGTGATGCG<br>R: CGGACACGGTCTGGGATA	RT-PCR
ACP79	F: CGGCTTGGGTCTCGGCGGCTAC<br>R: GCGGCTGCGATGCCAATG	RT-PCR
β-actin	F: CGAAGCACAGTCAAAGAGAGGTA<br>R: GCTTCAGTCAAGAGAACAGGATG	RT-PCR and RT-qPCR

Table S3 Statistics of annotation results

Annotation	Numbers
NR	26696
NT	12130
Swissport	21327
KEGG	18674
COG	9160
GO	13304
All	29289

Table S4 Summary of the identified cuticle proteins in *L.migratoria*

Unigenes ID	Family	ChtBD	Gene description
Unigene1463	RR-1	Y	Endocuticle structural glycoprotein (Abd-1)
Unigene1604	RR-1	Y	Endocuticle structural glycoprotein (Abd-2)
Unigene1553	RR-1	Y	Endocuticle structural glycoprotein (Abd-3)
Unigene1684	RR-1	Y	Endocuticle structural glycoprotein (Abd-4)
Unigene1562	RR-1	Y	Endocuticle structural glycoprotein (Abd-5)
Unigene1578	RR-1	Y	Endocuticle structural glycoprotein (Abd-6)
Unigene1904	RR-1	Y	Endocuticle structural glycoprotein (Abd-8)
Unigene1610	RR-1	Y	Endocuticle structural glycoprotein (Abd-9)
CL694.Contig1	RR-1	Y	early cuticle protein 2
CL694.Contig2	RR-1	Y	early cuticle protein 3
CL5853.Contig1	RR-1	Y	early cuticle protein 4
Unigene1033	RR-1	Y	putative cuticle protein CP5
Unigene1514	RR-1	Y	early cuticle protein 6
Unigene9827	RR-1	Y	larval cuticle protein 8
CL6188.Contig1	RR-1	Y	flexible cuticle protein 12-like
Unigene4267	RR-1	Y	cuticle protein 2
Unigene12826	RR-1	Y	Flexible cuticle protein 12
Unigene1613	RR-1	Y	larval cuticle protein 12.3
CL2845.Contig1	RR-1	Y	hypothetical protein YQE_08581
Unigene4928	RR-1	Y	AAEL013514-PA
Unigene1567	RR-1	Y	hypothetical protein TcasGA2_TC014498
CL3709.Contig6	RR-1	Y	larval cuticle protein LCP-17-like precursor
CL4965.Contig1	RR-1	Y	cuticular protein RR-1 motif 27 precursor
CL1330.Contig11	RR-1	Y	Cpr49Aa, partial
Unigene1513	RR-1	Y	cuticular protein RR-1 family member 53 precursor
Unigene3071	RR-2	Y	Adult cuticle protein 7 (ACP7)
CL7107.Contig2	RR-2	Y	Adult cuticle protein 8 (ACP8)
CL1285.Contig1	RR-2	Y	cuticle protein 7-like

Unigene1528	RR-2	Y	cuticle protein 18.6, isoform A-like
Unigene14515	RR-2	Y	cuticle protein 18.6, isoform B-like
Unigene3791	RR-2	Y	Adult cuticle protein 19 (ACP19)
CL2086.Contig2	RR-2	Y	Nymph cuticle protein 19.8 (NCP19.8)
Unigene13961	RR-2	Y	adult-specific cuticular protein ACP20
Unigene435	RR-2	Y	Adult cuticle protein 21 (ACP21)
CL5595.Contig1	RR-2	Y	adult-specific cuticular protein ACP22
Unigene5041	RR-2	Y	structural contituent of cuticle
CL7266.Contig2	RR-2	Y	Larval cuticle protein A3A
CL7266.Contig1	RR-2	Y	larval cuticle protein A2B-like
Unigene5040	RR-2	Y	cuticular protein 116, RR-2 family
Unigene3748	RR-2	Y	pro-resilin-like
Unigene486	RR-2	Y	Pro-resilin
Unigene1846	RR-2	Y	Pupal cuticle protein Edg-84A-like Protein
CL1363.Contig1	RR-2	Y	GH17178
CL4063.Contig1	RR-3	Y	Cuticle protein 6
Unigene2600	RR-3	Y	NCP14.9
Unigene3881	RR-3	Y	cuticular protein 3
Unigene23502	RR-3	Y	cuticular protein precursor
Unigene1788	RR-3	Y	cuticle protein, putative
Unigene1801	RR-3	Y	Pupal cuticle protein Edg-78E
Unigene3319	RR-3	Y	hypothetical protein YQE_02146
CL7584.Contig1	RR-3	Y	hypothetical protein TcasGA2_TC015304
Unigene1893	CPAP3	Y	LmObst-A1
Unigene1946	CPAP3	Y	LmObst-A2
Unigene2346	CPAP3	Y	LmObst-B
Unigene4275	CPAP3	Y	LmObst-C
Unigene1772	CPAP3	Y	LmObst-D1
Unigene2115	CPAP3	Y	LmObst-D2
Unigene4514	CPAP3	Y	LmObst-E1
Unigene3887	CPAP1	Y	LmObst-H
Unigene25974	CPAP1	Y	LmObst-F
CL6568.Contig1	Tweedle	Y	Tweedle 1
Unigene48534	Tweedle	Y	Tweedle 2
Unigene1519	CPF&CPFL	N	CPF1 (NCP4.9)
Unigene16603	CPF&CPFL	N	CPF2 (ACP38)
Unigene1530	CPF&CPFL	N	CPF3 (ACP63)
CL379.Contig2	CPF&CPFL	N	CPF4 (ACP64)
Unigene1529	CPF&CPFL	N	CPF5 (NCP21.3)
CL101.Contig2	CPF&CPFL	N	CPFL1 (NCP6.4)
Unigene3699	CPF&CPFL	N	CPFL2 (NCP1)

Unigene4714	CPF&CPFL	N	CPFL3 (ACP76)
CL4805.Contig1	CPF&CPFL	N	CPFL4 (ACP79)
Unigene496	Others	N	NCP9.5
Unigene1585	Others	N	NCP18.7
Unigene385	Others	N	NCP62
Unigene1724	Others	N	ACP16.5A
Unigene9168	Others	N	NCP3
Unigene1658	Others	N	proline-rich protein 4
CL1285.Contig4	Others	N	Cuticle protein 4
Unigene14788	Others	Y	neoplasm 1-like
Unigene3087	Others	Y	hypothetical protein BE21_11430
Unigene1647	Others	Y	cuticular protein 78
